# Autonomous Mission Planning for Fixed-Wing Unmanned Aerial Vehicles in Multiscenario Reconnaissance

**DOI:** 10.3390/s25041176

**Published:** 2025-02-14

**Authors:** Bei Chen, Jiaxin Yan, Zebo Zhou, Rui Lai, Jiejian Lin

**Affiliations:** 1 School of Aeronautics and Astronautics, University of Electronic Science and Technology of China, Chengdu 611731, China; 202112100618@std.uestc.edu.cn (B.C.); 202411100611@std.uestc.edu.cn (J.Y.); o1n1n7@gmail.com (J.L.); 2 AVIC (Chengdu) Unmanned Aerial Vehicle Systems Co., Ltd., Chengdu 611743, China; lairuihit@163.com; 3 System of Systems and Artificial Intelligence Laboratory, Chendu 610041, China

**Keywords:** fixed-wing UAV, mission planning, coverage path planning, payload characteristics

## Abstract

Before a fixed-wing UAV executes target tracking missions, it is essential to identify targets through reconnaissance mission areas using onboard payloads. This paper presents an autonomous mission planning method designed for such reconnaissance operations, enabling effective target identification prior to tracking. Existing planning methods primarily focus on flight performance, energy consumption, and obstacle avoidance, with less attention to integrating payload. Our proposed method emphasizes the combination of two key functions: flight path planning and payload mission planning. In terms of path planning, we introduce a method based on the Hierarchical Traveling Salesman Problem (HTSP), which utilizes the nearest neighbor algorithm to find the optimal visit sequence and entry points for area targets. When dealing with area targets containing no-fly zones, HTSP quickly calculates a set of waypoints required for coverage path planning (CPP) based on the Generalized Traveling Salesman Problem (GTSP), ensuring thorough and effective reconnaissance coverage. In terms of payload mission planning, our proposed method fully considers payload characteristics such as scan resolution, imaging width, and operating modes to generate predefined mission instruction sets. By meticulously analyzing payload constraints, we further optimized the path planning results, ensuring that each instruction meets the payload performance requirements. Finally, simulations validated the effectiveness and superiority of the proposed autonomous mission planning method in reconnaissance tasks.

## 1. Introduction

In recent years, fixed-wing UAVs have played an increasingly important role in various reconnaissance mission scenarios. Their unique flight performance and long endurance capabilities have demonstrated significant application potential in both military reconnaissance and civilian monitoring fields [1,2,3]. One crucial application is target tracking [4,5,6]. Before a fixed-wing UAV executes target tracking missions, it is crucial to first identify targets within reconnaissance mission areas using onboard payloads. This paper presents an autonomous mission planning method designed for these reconnaissance operations, enabling effective target identification prior to initiating tracking tasks. We believe that UAVs completing reconnaissance missions must address two main aspects: path planning and payload planning. However, traditional planning methods are often limited to single-objective optimization, such as focusing on flight performance, obstacle avoidance, and energy consumption [7,8,9], while neglecting the deep integration of payload characteristics.

Currently, UAV path planning works are primarily focused on applications with single scenarios, lacking consideration of different scenarios. Cui [10] formulated the problem of multiple single-point reconnaissance targets as a Dynamic Constrained Traveling Salesman Problem with Neighborhoods (DCTSPN) and proposed a hierarchical algorithm based on deep reinforcement learning to solve it. Although the effectiveness of this algorithm was validated, it is well known that learning-based methods are not interpretable. Zhang et al. [11] proposed a bi-level hybridization-based metaheuristic algorithm for fixed-wing UAVs used in forest fire monitoring. While this algorithm can reduce data collection cycles and energy consumption in most cases, it is limited to single-point targets, restricting its applicability to other scenarios. At the same time, the coverage path planning (CPP) problem has garnered increasing attention [12,13,14,15], especially in missions requiring UAVs to scan entire areas. The CPP problem involves planning a path that covers all Regions of Interest (ROI) while avoiding obstacles or no-fly zones [16,17,18]. Different CPP methods have their own advantages and limitations. Selecting an appropriate CPP method requires the consideration of specific application requirements and environments [19]. Barrientos et al. [20] proposed a Breadth First Search (BFS)-based coverage planner, which can be applied to grid-based workspaces to generate paths with minimal turning maneuvers. However, search-based methods have high computational complexity, particularly in large-scale environments where they require substantial computational resources, making them unsuitable for long-endurance fixed-wing UAVs. In [21], a random walk-based CPP algorithm for robots was introduced. This method is simple and has low memory requirements, making it easy to deploy. However, random walk paths are only suitable for smaller environments and struggle to cover ROI with obstacles. Additionally, the robot may traverse the same path multiple times, leading to overall inefficiency in the coverage path. Bähnemann et al. [22] proposed a decomposition-based CPP algorithm for quadrotor, which extends the Boustrophedon Cell Decompositon (BCD) [23,24,25] by optimizing different sweep combinations to find the optimal sweep path and accounting for obstacles between the decomposed cells. This method is computationally efficient, easy to implement, and effectively handles complex areas.

Although the above works have achieved significant results in reconnaissance path planning for single-point or ROI targets, few works have been able to combine these two typical scenarios. Additionally, these studies have not adequately considered the role of payloads in the planning process. We need to clearly recognize that path planning without effective payload used for reconnaissance integration cannot ensure the successful execution of flight missions [26,27,28,29]. We introduce a reconnaissance fixed-wing UAV, typically operating at altitudes above 5000m, equipped with Synthetic Aperture Radar (SAR) systems for aerial reconnaissance [30,31]. SAR systems have two operating modes: beam forming [32] and strip modes [33]. One of the most challenging aspects of mission planning is adjusting the UAV’s path based on the payload. This complexity arises from the need to combine the payload’s system response time, imaging width, and others with the existing flight plan. Such integration is crucial for meeting mission requirements.

Therefore, this paper takes this as a motivation to explore how to achieve autonomous mission planning for fixed-wing UAVs in multiscenario reconnaissance. The innovations of this paper include the following: Firstly, in Section 2, we designed a path planning algorithm featuring the HTSP. This algorithm initially solves the TSP problem using the nearest neighbor algorithm to determine the reconnaissance sequence for different mission targets. Additionally, by incorporating the BCD technique, it addresses the coverage problem for area targets. We transformed the GTSP problem into a graph structure, ensuring the accuracy of the planning results. Fixed-wing UAVs need to switch flexibly between different mission scenarios. In Section 3, the proposed payload planning module considers the characteristics, workflow, and instruction parameters of the payload. It adjusts the pre-planned waypoint set according to the payload’s operating mode. Furthermore, to ensure the safety of UAV transitions between distinct mission scenarios, a visibility graph obstacle avoidance algorithm was integrated to refine the waypoint sets. Finally, in Section 4, we developed a user-friendly UI interface that supports visualization and adjustment, and validated the effectiveness of the autonomous mission planning module in multiscenario situtations.

## 2. HTSP Path Planning

In multiscenario situations, UAVs must visit multiple point targets, which are specific locations, and area targets, which are ROI with defined boundaries. This problem can be modeled as an HTSP. The main challenge of HTSP is to determine the sequence of visiting each target to ensure the shortest flight path, as well as to plan the coverage flight path within each area target.

To address the HTSP problem, we adopt a two-stage strategy:

1. Sequence Planning: This stage determines the order of visiting different scenarios. By optimizing the sequence, the UAV can minimize the overall flight range.
2. Coverage Path Planning: The lower level addresses the coverage of area targets using BCD. This ensures that the UAV efficiently covers each area while adhering to constraints such as no-fly zones.

### 2.1. Sequence Planning

The inputs for the autonomous mission planning module include the entry point S, the exit point E, the set of no-fly zones or obstacle Obs, the set of area target F, and the set of point targets M.

(1) $$\left\{\begin{array}{c} S=\{x_{S},y_{S},h_{S}\}\\ E=\{x_{E},y_{E},h_{E}\}\\ \mathrm{Obs}=\{{\mathrm{obs}}_{1},{\mathrm{obs}}_{2},\ldots ,{\mathrm{obs}}_{i}\}\\ {\mathrm{obs}}_{i}=\left\{op_{i1},op_{i2},\ldots ,op_{in}\right\}\\ op_{in}=\left\{x_{in},y_{in},h_{in}\right\}\\ F=\{f_{1},f_{2},\ldots ,f_{j}\}\\ f_{j}=\left\{\left\{fv_{j1},fv_{j2},\ldots ,fv_{jn}\right\},mode,iw\right\}\\ fv_{jn}=\left\{x_{jn},y_{jn},h_{jn}\right\}\\ M=\{m_{1},m_{2},\ldots ,m_{k}\}\\ m_{k}=\left\{x_{k},y_{k},h_{k},mode,iw\right\} \end{array}\right.$$

In (1), *x*, *y*, and *h* represent the two-dimensional coordinates and altitude, respectively. ${\mathrm{obs}}_{i}$ denotes the no-fly zones constructed as polygons, with their vertices represented by $op_{in}$. $f_{j}$ represents the area targets, also constructed as polygons, with their vertices represented by $fv_{jn}$. $m_{k}$ refers to the point targets. mode and iw are integer variables indicating the payload operating mode and imaging width, respectively.

Sequence planning uses the nearest neighbor algorithm not only to determine the traversal order but also to further output the entry points for the coverage paths of polygonal area targets. Identifying the entry points for area targets helps minimize the overall path length. When covering polygons, choosing entry points judiciously can prevent redundant paths, thereby reducing the total flight range. The point set *V* is constructed as follows:

(2) $$V=\left(\overset{m}{\underset{n=1}{⋃}}fp_{jn}\right)\cup \left(\overset{l}{\underset{k=1}{⋃}}M_{k}\right)\cup \left\{S,E\right\}$$

where *m* represents the number of area targets and *l* represents the number of point targets. To compute the Euclidean distance between any two points in the set *V*, we obtain the distance matrix *D*:

(3) $$D_{ij}=\sqrt{{\left(x_{i}-x_{j}\right)}^{2}+{\left(y_{i}-y_{j}\right)}^{2}}$$

where $\left(x_{i},y_{i}\right)$ and $\left(x_{j},y_{j}\right)$ are the coordinates of points $V_{i}$ and $V_{j}$, respectively. Using the nearest neighbor algorithm, we solve for the path from the entry point *S* to the exit point *E* that passes through all points, denoted as $T_{seq}$. We initialize the current node as $current=v_{0}$ and the starting point as $v_{0}=S$. We initialize a boolean array visited to mark the visit status of all nodes, where all nodes are initially unvisited: visited={false,false,…,false}. We mark the starting node $v_{1}$ as visited: visited[0]=true). We initialize the sequence $T_{\mathrm{seq}}$ and add the starting node to the sequence: $T_{\mathrm{seq}}=\left[v_{1}\right]$.

We loop from the starting node to the second-to-last node, executing the following iterative steps: For each node *i* from 1 to n−2, we select the node next closest to node current among all unvisited nodes, and thus we have:

(4) $$next=\arg\min_{j\notin \mathrm{visited}}D_{current,j}$$

We add next to the sequence:

(5) $$T_{seq}=T_{seq}\cup \left\{next\right\}$$

We update the current node.

(6) current=next

We mark node next as visited.

(7) visited[next]=true

Finally, we add the exit point $v_{n-1}=E$ to the sequence.

(8) $$T_{seq}=T_{seq}\cup \left\{v_{n-1}\right\}$$

From Equations (4)–(8), we use the nearest neighbor algorithm to obtain the shortest sequence of point set $T_{seq}$ from S to E.

Finally, we determine the entry point of each polygon based on $T_{seq}$. We initialize the entry point array for each polygon entry_points:

(9) $$entry\_points={\left\{\left(0,0\right)\right\}}_{i=1}^{m}$$

We initialize the polygon visit array poly_visited:

(10) $$poly\_visited={\left\{\mathrm{false}\right\}}_{i=1}^{m}$$

We iterate over each element $V_{i}$ in the sequence $T_{seq}$. If $V_{i}$ belongs to the *i*-th polygon and this polygon has not yet been visited, then we set $V_{i}$ to:

(11) $$entry\_points\left[i\right]=V_{i}$$

We mark the *i*-th polygon as visited.

(12) polygon_visited[i]=true

Through the formalization shown above, we can comprehensively describe the nearest neighbor algorithm for solving the approximate TSP problem, ensuring that the shortest sequence of target visits and entry points is determined from the start S to the end E.

### 2.2. Coverage Path Planning with Area Target

For UAVs equipped with SAR systems, continuous scanning is performed along the coverage path during a single pass over the area target. The data collected are processed by algorithms to generate high-resolution images. However, suboptimal flight paths can significantly degrade image quality. To ensure optimal scanning, the planning of a UAV’s coverage path becomes even more critical.

To address the coverage path planning problem, the module employs the BCD algorithm to divide area targets with no-fly zones into multiple cells. This decomposition ensures that each cell is a simple polygon free of no-fly zones.

BCD is a method used to divide an AOI into smaller, simpler cells that can be covered systematically. The main goal is to ensure the complete coverage of the area while avoiding obstacles, ensuring efficient traversal and minimizing the total path length.

Let us consider an AOI $f_{j}$ represented as a polygon with vertices $\left\{fv_{j1},fv_{j2},\ldots ,fv_{jn}\right\}$. The AOI may contain no-fly zones Obs, each represented as a polygonal region. The task is to cover the area $f_{j}$ while avoiding these no-fly zones. The algorithm identifies critical points on the boundaries of $f_{j}$ and Obs, which are points where the sweep line direction changes. This could be the vertices of $f_{j}$ and Obs. A sweep line moves from left to right (or top to bottom), dividing the area into cells whenever it encounters a critical point. The cells are simple polygons, free from any no-fly zones.

Each cell $C_{j}k$ is formed as a result of the sweep line passing through critical points. Mathematically, a cell $C_{j}k$ is represented as:

(13) $$C_{j}k=f_{j}\setminus \overset{m}{\underset{i=1}{⋃}}{\mathrm{obs}}_{i}$$

where $C_{j}k$ is a simple polygon without holes, and *m* is the number of obstacles in the AOI.

Within each cell, a systematic back-and-forth (Boustrophedon) motion is used to cover the entire area. This motion can be described mathematically by generating parallel lines inside the cell, ensuring that they cover the cell fully without overlapping.

We choose a direction for the parallel lines. This is often chosen as perpendicular to the longest edge or the edge that aligns best with the general shape of the cell to minimize the number of turns. Let this direction be denoted by a unit vector d.

The coverage path within $C_{j}k$ is generated as a set of parallel lines $l_{k}i$ that are spaced apart by the offset Δf. These lines are perpendicular to the chosen direction d. Mathematically, $l_{k}i$ can be represented as:

(14) $$l_{k}i:r=r_{0}+i\Delta fd^{⊥},\;i=0,1,2,\cdots ,n$$

where r is the position vector of a point on the line. $r_{0}$ is the position vector of the starting point of the first line $L_{0}$, typically taken as the intersection of the first line with the boundary of $C_{j}k$. These lines are separated by a distance called offset, denoted as Δf. $d^{⊥}$ is a unit vector perpendicular to d. *n* is the number of parallel lines required to cover the entire cell.

For each line segment $l_{k}i$, we identify the start and end points:

(15) $$P_{ki}=\left\{p_{ki}^{start},p_{ki}^{end}\right\},\;i=0,1,2,\ldots ,n$$

Consequently, we obtain a set of all waypoints for the UAV to cover $C_{j}k$, which can be represented as:

(16) $$P_{j}k=\left\{P_{k1},P_{k2},\ldots \right\}$$

To minimize the total path length of the UAV, the coverage paths between adjacent cells are optimized. The objective is to find the optimal sequence for visiting cells, ensuring that each cell is visited exactly once. This problem is typically addressed using graph-based algorithms, and it is modeled as a GTSP.

The GTSP formulation [34,35] for UAV coverage path planning is defined as follows:

(17) $$\mathrm{Min}\sum_{i=1}^{m}\sum_{j=1}^{m}d_{ij}x_{ij}$$

where $d_{ij}$ represents the travel distance between cell *i* and cell *j*. $x_{ij}$ is a binary decision variable indicating whether the UAV travels directly from cell *i* to cell *j*. *N* is the set of all cells.

The optimization is subject to the following constraints:

Constraints:

1. Binary decision variables:
  (18) $$x_{ij}\in \left\{0,1\right\}\;\forall i,j$$
2. We set the start *O* to the cell where the entry point *E* obtained in Section 2.1 is located.
  (19) $$\sum_{i\in V}x_{Oi}=1$$
  *V* is the set of cells except *O*.
3. We ensure that each cell is entered and left at most once:
  (20) $$\sum_{i\in N}x_{ij}\leq 1\;\forall j\in V$$
  (21) $$\sum_{j\in N}x_{ij}\leq 1\;\forall i\in V$$
4. Subtour elimination constraints (Miller–Tucker–Zemlin formulation):
  (22) $$u_{i}-u_{j}+m\cdot x_{ij}\leq m-1\;\forall i,j\in V,i\neq j$$
  (23) $$1\leq u_{i}\leq m-1\;\forall i\in V$$
  where $u_{i}$ is an auxiliary variable which keeps the number of visited cells until cell *i* in the solution. These constraints ensure that the solution forms a single continuous path.

We solve the GTSP using GLKH (http://webhotel4.ruc.dk/~keld/research/GLKH/, accessed on 5 November 2024) as an open source solver [36]. After solving the GTSP, the coverage waypoints $P_{j}$ of visiting the area target $f_{j}$ are obtained. From the sequence planning in the previous section, we obtain the access order of the target $T_{seq}$. Let $T_{seq}=\left\{T_{1},T_{2},\cdots ,T_{n}\right\}$ represent the sequence in which the targets are accessed, where each $T_{i}$ is either a point target or an area target. $P_{T}$ represents the waypoints associated with target *T*. For point target $m_{k}$, the waypoint set $P_{k}$ may consist of a single waypoint. For area target $f_{j}$, the waypoint set $P_{j}$ consists of multiple waypoints that cover the entire surface area. In summary, the pre-planned waypoints *P* of the HTSP are obtained by connecting the waypoints of each target $T_{i}$ in the sequence $T_{seq}$:

(24) $$P=\overset{n}{\underset{i=1}{⋃}}P_{T_{i}}$$

## 3. Payload Mission Planning

**Figure 1:**
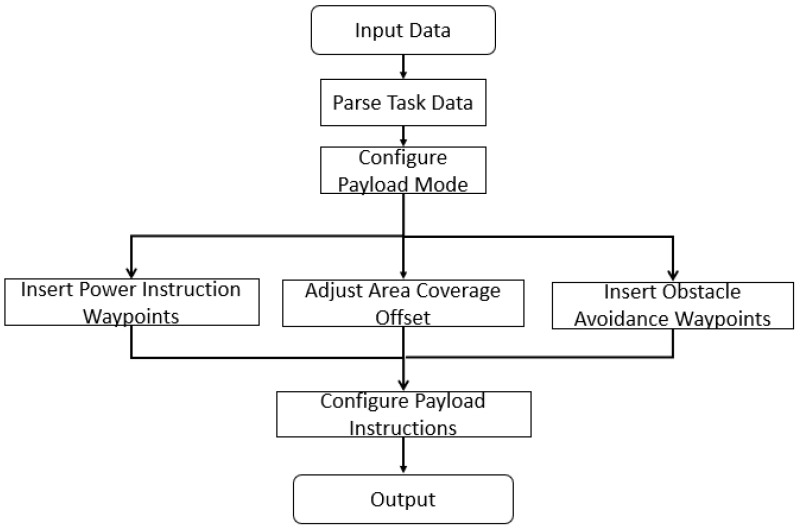
Computational workflow for UAV payload mission planning.

In reconnaissance missions, the performance and efficiency of the UAV’s payload are paramount. The payload mission planning process must therefore account for the specific operational characteristics and constraints. This section integrates payload characteristics to adjust the pre-planned waypoints *P*. Additionally, obstacle-avoidance waypoints during UAV cruising are generated based on a visibility graph method. The computational workflow for this section is designed to optimize the integration of payload characteristics and waypoint adjustments, as outlined in Figure 1.

Equation (1) is the input for the autonomous mission planning module. It will provide the required payload mode (e.g., SAR Beam Focusing or SAR Strip) and iw for each target. We will complete mission planning based on this information.

### 3.1. Power Instruction

The first step in payload mission planning is managing the power state of the payload, which includes turning it on, standby, etc., depending on the mission’s requirements. The goal here is to ensure that the payload is fully operational when the UAV reaches the target area, thereby avoiding any delays or inefficiencies in data acquisition.

Assuming that the payload’s power-on time is $t_{on}$ with the input mode in (1) and the UAV’s speed is *v*, we calculate the distance $d_{on}^{i}$ from the target $T_{i}$ where the power-on command should be issued:

(25) $$∥p_{on}-p_{i}∥\geq d_{on}=t_{on}\times v$$

In this formulation, $p_{on}$ is the waypoint that needs to be inserted, and $p_{i}$ is the position of $T_{i}$, which may be the point target position or the entry position of an area target. The updated set of waypoints *P* after inserting $p_{on}$ can be expressed as:

(26) $$P^{\prime }=\left\{P_{T_{1}},P_{T_{2}},\ldots ,p_{on},P_{T_{i}},\ldots ,P_{T_{n}}\right\}$$

Similarly, other kinds of commands for the payload can be inserted into the pre-planned waypoint set in the same manner, which will not be elaborated further here. By ensuring that the power command is issued at the correct waypoint, the UAV’s payload will be in the appropriate operational state upon reaching the target.

### 3.2. Coverage Path Optimization with Payload

Different operating modes of the UAV’s payload have different imaging widths based on factors like resolution and scan mode. By adjusting the offset of the coverage path to match the specific imaging width of the payload in its current mode, we ensure that each pass of the UAV maximizes its coverage area. This minimizes the number of required passes, thereby reducing mission time and energy consumption while ensuring complete coverage without gaps or excessive overlaps.

To adjust the offset of the full coverage path of $f_{j}$, we need to replace the offset Δf in Equation (14) with the effective iw of the payload.

We adjust Δf to iw, so the updated formula for the coverage path lines $l_{ki}$ becomes:

(27) $$l_{ki}:r=r_{0}+iw_{iw}d^{⊥},\;i=0,1,2,\ldots ,n$$

Based on Equations (14)–(24), we can obtain the adjusted coverage waypoint set ${P_{T_{j}}}^{\prime }$ for $f_{j}$. The modified coverage waypoints are then inserted into the pre-planned waypoint set *P*, as shown in the following equation:

(28) $$P^{\prime }=\left\{P_{T_{1}},P_{T_{2}},\ldots ,{P_{T_{j}}}^{\prime },P_{T_{j}+1},\ldots ,P_{T_{n}}\right\}$$

The payload mission planning process described in this section plays a crucial role in the overall mission planning for reconnaissance fixed-wing UAVs. By carefully managing the power and optimizing the coverage path based on the payload’s characteristics, a UAV can achieve optimal reconnaissance performance, ensuring that all mission objectives are met efficiently and effectively.

### 3.3. Obstacle Avoidance Based on Visibility Graph

**Figure 2:**
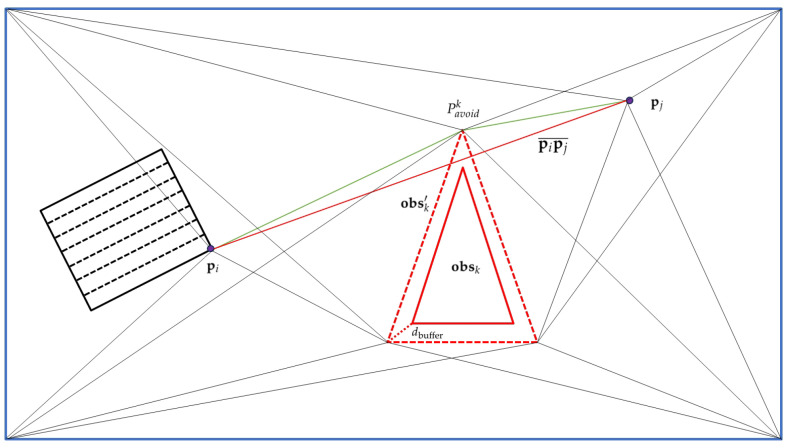
Visualization of the visibility graph algorithm for UAV obstacle avoidance. The algorithm identifies potential intersections between the planned path segment $\bar{p_{i}p_{j}}$, represented by the red line segment, and obstacle polygons. Obstacles are expanded by a buffer distance $d_{\mathrm{buffer}}$ to ensure safety, and alternate paths, such as through $p_{\mathrm{avoid}}^{k}$, are generated to bypass obstacles, as illustrated by the green line.

Regardless of whether the target is a point or an area, obstacles may still exist in the region between two targets. Therefore, an obstacle avoidance method based on visibility graphs is adopted. The key steps of the visibility graph algorithm are as shown in Figure 2:

Firstly, each obstacle is represented as a convex polygon. For non-convex obstacles, convex decomposition is performed to divide them into multiple convex components. Subsequently, the vertices of the obstacle polygons are expanded by a safety buffer distance $d_{\mathrm{buffer}}$ to account for the UAV’s size and maneuvering limitations:

$${\mathrm{obs}}_{k}^{\prime }=\left\{{\mathrm{op}}_{kn}+d_{\mathrm{buffer}}n_{kn}|{\mathrm{op}}_{kn}\in {\mathrm{obs}}_{k}\right\}$$

Here, $n_{kn}$ represents the outward normal vector at vertex ${\mathrm{op}}_{kn}$.

Next, for the last waypoint $p_{i}$ of the preceding target and the first waypoint $p_{j}$ of the next target in the planned path, we examine whether the line segment $\bar{p_{i}p_{j}}$ intersects with any obstacles. The intersection condition can be expressed as:

$$\bar{p_{i}p_{j}}\cap {\mathrm{obs}}_{k}^{\prime }\neq \emptyset \;\forall k$$

The visibility graph G=(V,E) is constructed where *V* contains all obstacle vertices, $p_{i}$ and $p_{j}$. *E* contains edges between vertices that are mutually visible.

Two vertices $v_{i}$ and $v_{j}$ are mutually visible if:

(29) $$\bar{v_{i}v_{j}}\cap {\mathrm{obs}}_{k}^{\prime }=\emptyset \;\forall k$$

Then, using Dijkstra’s algorithm on the visibility graph, we find the shortest collision-free path between $p_{i}$ and $p_{j}$:

(30) $$P_{avoid}^{k}=\arg\min_{p\in G}\sum_{i=1}^{n-1}∥v_{i+1}-v_{i}∥$$

Finally, the obstacle avoidance waypoints are integrated into the final path:

(31) $$P_{final}=\left\{P_{T_{1}},P_{T_{2}},\ldots ,P_{T_{k}},P_{avoid}^{k},P_{T_{k}+1},\ldots ,P_{T_{n}}\right\}$$

This approach ensures safe navigation while maintaining optimal path efficiency. The visibility graph method is particularly effective in environments with multiple convex obstacles, providing guaranteed collision-free paths with minimal computational overhead.

## 4. Experiments and Discussions

The experimental platform for algorithm validation consisted of a high-performance computing workstation, assembled by Lenovo in Beijing, China. The workstation was equipped with an AMD Ryzen 9 7945HX CPU (16 cores, 2.5 GHz base frequency) and 16 GB DDR4 RAM. The computational environment was implemented in Visual Studio 2022 running on Windows 11. All algorithms were implemented using the Computational Geometry Algorithms Library [37] (CGAL, https://doc.cgal.org/latest/Manual/index.html, accessed on 5 November 2024) in C++, which provides robust implementations of geometric algorithms including polygon operations, convex hulls, and visibility graphs that are essential for our path planning and obstacle avoidance computations. The user interface was developed using the QT framework, providing an interactive visualization environment for mission planning and results analysis. All data were executed on this platform to ensure consistent and reliable performance metrics.

### 4.1. UI Design

**Figure 3:**
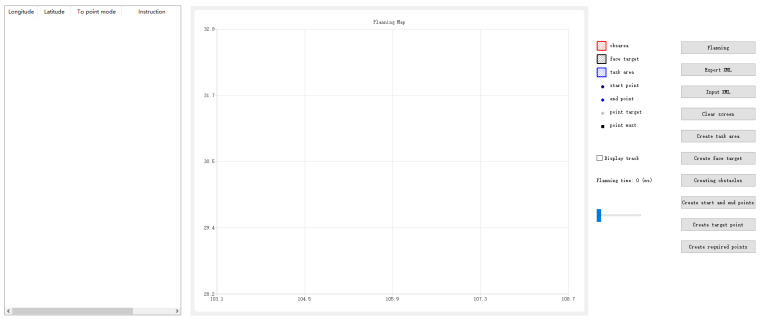
UI for autonomous mission planning module.

We developed a user-friendly interface for the autonomous mission planning module, as shown in Figure 3, organized into three functional areas from right to left. The right section features input controls for defining mission areas, targets, and no-fly zones, along with options to manage mission parameters such as speed and altitude, and to import/export planning data in XML format. The central section displays a dynamic map with latitude and longitude axes, visually representing the mission area, targets, no-fly zones, and the UAV’s planned waypoints. The left section presents a detailed table of the mission’s waypoints, including their sequence, coordinates, UAV arrival modes, and payload control instructions. This interface streamlines mission setup, visualization, and result analysis.

### 4.2. Autonomous Mission Planning

**Figure 4:**
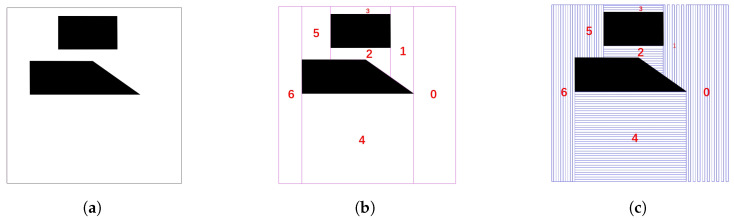
Area target with no-fly zones and its Boustrophedon Cell Decomposition. (**a**) An area target with no-fly zones. (**b**) Boustrophedon Cell Decomposition of an area target with no-fly zones.The area target is decomposed into 6 cells without no-fly zones, marked from 0 to 6 respectively. (**c**) Results of coverage path planning with an area target.

BCD stands out for its ability to ensure complete coverage of the area while simplifying the path planning problem. By dividing the workspace into simple, non-overlapping cells, BCD transforms the complex environment into a set of smaller polygons, each free of obstacles. This decomposition not only reduces computational complexity but also enhances the efficiency of path planning algorithms, ensuring that the UAV can systematically cover the entire area with minimal redundant traversal. Figure 4 visually represents this process, highlighting how the region is decomposed into discrete cells around no-fly zones.

In the experimental results depicted in Figure 4a, the BCD method was applied to an area target defined by the vertices (50,50), (50,800), (800,800), and (800,50), which encompasses two polygonal no-fly zones with vertices (148,275), (418,275), (621,148), (148,418) and (270,82), (523,82), (523,224), (270,224). These no-fly zones are represented as black polygons within the diagram. The BCD method successfully divided the complex area into seven distinct cells (marked from left to right), each free of no-fly zones. The decomposition process strategically navigated around the no-fly zones. This not only maintains the integrity of the no-fly zones but also maximizes the coverage of the area target by creating well-defined, accessible regions for the UAV to traverse.

Assuming the entry point of the area target in Figure 4b is the top left vertex, the optimal order for accessing these cells based on GTSP is as follows: 6→4→0→1→2→5→3. Assuming Δf=10, the result of the CPP based on BCD is shown in Figure 4c. Through the above numerical experiments, we have validated its effectiveness.

In the validation phase of the autonomous mission planning module, let us assume a fixed-wing UAV speed of 150 km/h and a flight altitude of 5000 m. The reconnaissance payload requires 70 s from power-on to operational state. The payload has two modes: an SAR Beam Focusing mode and an SAR Strip mode. For the SAR Beam Focusing mode, there are three resolution levels corresponding to three imaging widths, as shown in Table 1. Similarly, the characteristics of the SAR Strip mode are shown in Table 2. To better demonstrate the obstacle avoidance capability of the module in a large-span mission area, we set the $d_{\mathrm{buffer}}$ to 2000 m. The input target information includes the required reconnaissance payload mode and resolution for the mission. All position information is represented in latitude and longitude, and the coordinate transformation process during planning will not be elaborated here.

**Figure 5:**
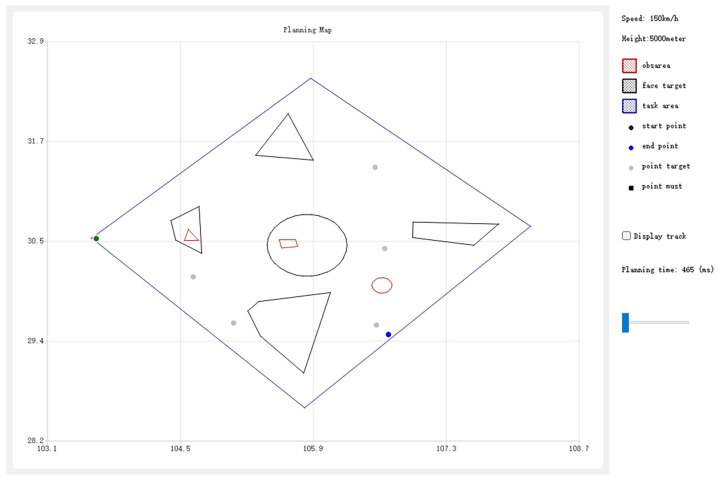
Planning map.

As shown in Figure 5, in the planning map, within a 300 km × 300 km mission area (blue polygon), we input five area targets (black polygons), three no-fly zones (red polygons), five point targets (gray points), the starting point (green point), and the endpoint (blue point).

**Figure 6:**
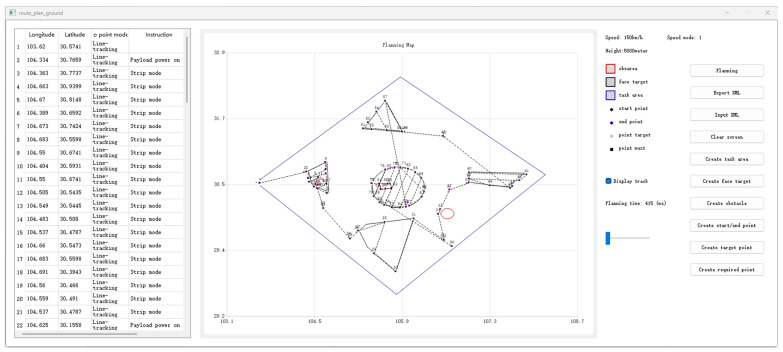
Planning results of autonomous mission planning module.

The proposed module was experimentally validated using the SAR Strip mode.The experiment was divided into two different scenarios. Figure 6 demonstrates the planning results. Firstly, sequence planning calculates the optimal access order, then mission planning optimizes the coverage path based on payload characteristics, and, finally, power instructions are also considered in the final mission instruction set. The following text will analyze these experimental results.

**Figure 7:**
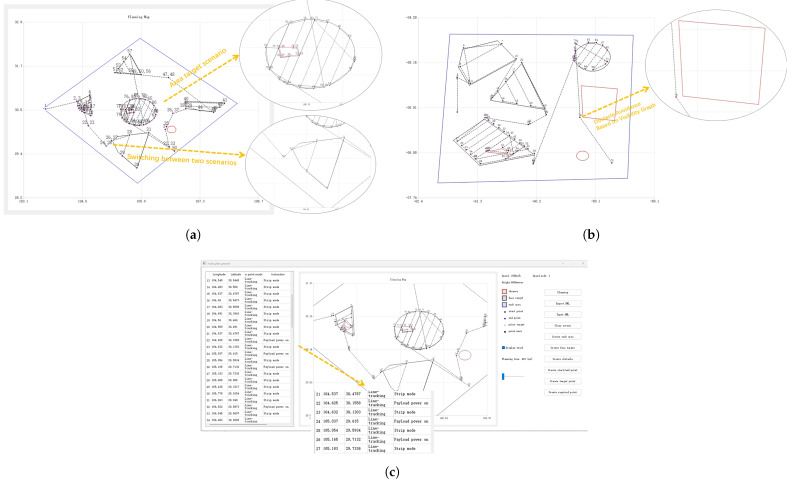
Path planning analysis, visibility graph-based obstacle avoidance, and payload power instruction output. (**a**) Path planning for different target scenarios, illustrating the planned waypoint set under varying conditions. (**b**) Visibility graph-based obstacle avoidance, demonstrating the generated path that navigates around no-fly zones and obstacles. (**c**) The output of payload power instructions, showing the power-on and work instructions of the payload during the mission. (**a**) Path planning for different targets. (**b**) Visibility graph-based obstacle avoidance for path planning. (**c**) The output of payload power instructions.

In the first area target scenario, the module successfully adjusted the UAV’s path to accommodate the different resolution requirements of five area targets. For targets 1, 3, and 5, which required high-resolution imaging (0.5), the UAV followed a finer, closer-offset path to ensure detailed coverage. Target 2, requiring medium resolution (1.0), had a lower path density. For target 4, with the lowest resolution requirement (3.0), the module optimized the path for broader coverage with minimal overlap. In the second scenario, the experiment focused on processing point target scenes, where the resolution requirement for all five point targets was uniformly set to 0.5. This scenario was mainly used to verify the module’s switching in various mission scenarios. Figure 7a illustrates the module output path. For the area target scenario, the module successfully adapts to varying resolution requirements, while the point target scenario demonstrates the module’s effective switching between scenarios. Figure 7b illustrates an obstacle avoidance method based on visibility graphs. By expanding obstacle vertices according to the $d_{\mathrm{buffer}}$, a visibility graph is constructed with waypoint No. 73 (lon: −59.5076; lat: −34.7782) as the starting point and waypoint No. 75 (lon: −58.8323; lat: −37.0782) as the endpoint. Utilizing Dijkstra’s algorithm, the shortest collision-free path is identified, successfully generating waypoint No. 74 (lon: −59.3972; lat: −36.1817) as the obstacle avoidance waypoint. The distance between this waypoint and the closest vertex of the rectangular obstacle (longitude: −59.368118; latitude: −36.192308) satisfies the requirement $d_{\mathrm{buffer}}$. This method ensures safe and efficient navigation.

**Figure 8:**
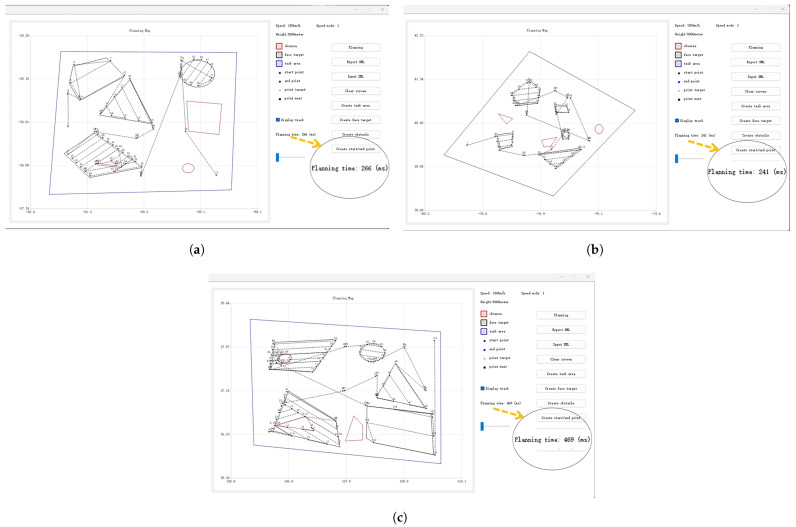
Comparison of algorithm execution times under three different test datasets. The results demonstrate that the computation time is within 0.5 s. (**a**) Time consumption results of the first set of test data. (**b**) Time consumption results of the second set of test data. (**c**) Time consumption results of the third set of test data.

As shown in the mission instruction list on the left side of Figure 7c, the module issued instruction No. 25 for the reconnaissance. Notably, the module inserted instruction No. 24 to power on the payload in advance, ensuring that it was fully operational upon reaching the target. To validate the computational efficiency of the proposed algorithm, we conducted multiple groups of test experiments. Each dataset consisted of 10 targets and three obstacles distributed across a 300 km × 300 km mission area. As shown in Figure 8, the experimental results demonstrate that the proposed method consistently completed calculations within 0.5 s across all test scenarios. The computation time, consistently below 500 ms, ensures compliance with the stringent operational requirements of fixed-wing UAVs.

## 5. Conclusions

This paper presented a comprehensive approach to autonomous mission planning for fixed-wing UAVs, specifically tailored to multiscenario reconnaissance missions. By addressing the limitations of existing UAV planning methods, which often overlook the integration of payload characteristics, we developed an advanced autonomous mission planning module that combines flight path planning and payload planning.

The proposed path planning strategy, grounded in the HTSP, effectively determines the optimal sequence for accessing reconnaissance targets and planning entry waypoints. The GTSP further enhances the coverage path planning, ensuring thorough reconnaissance even in complex environments with no-fly zones.

Our payload mission planning methodology meticulously considers key payload parameters such as scan resolution, imaging width, and modes. This ensures that each mission instruction is optimized to meet the payload’s performance requirements, thereby maximizing reconnaissance effectiveness.

Experiments demonstrated the practical applicability of the module, allowing for intuitive verification and validation of its effectiveness. Overall, the results affirm the superiority of our integrated mission planning module, providing a robust solution for efficient and effective UAV reconnaissance in diverse scenarios. Future work could extend this framework to accommodate additional mission constraints and enhance real-time adaptability in dynamic environments.

## Figures and Tables

**Table 1:** The characteristics of the SAR Beam Focusing mode.

Default Work Altitude	Imaging Resolution	Range	Imaging Width
5000 m	0.5 m	16–80 km	5 km × 5 km
	0.3 m	12–60 km	3 km × 3 km
	0.1 m	12–30 km	1 km × 1 km

**Table 2:** The characteristics of the SAR Strip mode.

Default Work Altitude	Imaging Resolution	Range	Imaging Width
5000 m	5 m	72–120 km	405 km
	1 m	60–100 km	203 km
	0.5 m	48–80 km	12 km

## Data Availability

Data are contained within the article.
